# Discovery of Deer Antler-Derived Antioxidant Peptides Through Computational and Cell-Based Approaches

**DOI:** 10.3390/antiox14101169

**Published:** 2025-09-25

**Authors:** Yongxin Jiang, Jingxian Zheng, Yan Zhang, Yuyang Liu, Linlin Zeng, Weiwei Han

**Affiliations:** Key Laboratory for Molecular Enzymology and Engineering of Ministry of Education, Edmond Fischer Cell Signaling Laboratory, School of Life Sciences, Jilin University, Changchun 130012, China; jiangyx23@mails.jlu.edu.cn (Y.J.); jxzheng24@mails.jlu.edu.cn (J.Z.); zyan22@mails.jlu.edu.cn (Y.Z.); liuyy1319@mails.jlu.edu.cn (Y.L.)

**Keywords:** antioxidant, deer antler peptides, Keap1, molecular dynamics simulations, virtual hydrolysis

## Abstract

Oxidative stress, caused by excessive free radicals, leads to cellular damage and various diseases. Antioxidant peptides from natural proteins offer potential in alleviating this stress. In this study, antioxidant peptides were identified from deer antler proteins using in silico enzymatic hydrolysis and machine learning. Peptides with high prediction scores and non-toxic profiles were selected for evaluation. The antioxidant activities of top candidates, PHPAPTL and VPHGL, were confirmed by radical scavenging assays and their protective effects in HepG2 cells. Molecular dynamics simulations revealed stable binding of these peptides to Keap1, enhancing system stability and reducing residue fluctuations at the ligand-binding interface. Key interactions involved Arg415, Arg483, Arg380, and Ser555. Secondary structure analysis showed peptide binding induced local conformational transitions, notably increasing parallel β-sheet formation near active sites. These findings provide mechanistic insight into their antioxidant effects and support their potential application in functional food development.

## 1. Introduction

Free radicals are molecules containing one or more unpaired electrons, characterized by high chemical reactivity. They are generated both during normal metabolic processes and under pathological conditions or external stimuli. For instance, reactive oxygen species (ROS), a type of free radical, are produced as byproducts of normal metabolism in the mitochondrial electron transport chain, serving as essential components in maintaining physiological homeostasis. However, exposure to factors such as inflammation, toxic chemicals, or radiation can trigger excessive free radical production [1]. When the generation of free radicals surpasses the capacity of the body’s antioxidant defense system, oxidative stress occurs, leading to cellular damage and contributing to the development of various diseases, including cancer [2], cardiovascular diseases [3], and neurological disorders [4]. Therefore, timely supplementation with antioxidants capable of scavenging free radicals is of great significance for maintaining human health.

In the search for natural antioxidants, traditional medicinal resources have attracted considerable attention. Velvet antler, a valuable traditional medicinal material, has been used as a health supplement and therapeutic agent in China, Japan, and Korea for thousands of years. It has demonstrated potential in the prevention and treatment of multiple diseases, such as osteoporosis [5], depression [6], and breast cancer [7]. Bioactive peptides, composed of short chains of amino acids with specific physiological regulatory functions, generally exhibit higher bioactivity than intact proteins, mainly due to the greater exposure of their functional groups [8]. Among them, antioxidant peptides—a class of functional small molecules—can scavenge free radicals and alleviate oxidative stress, offering promising applications in anti-aging and the prevention of various chronic diseases. Velvet antler-derived peptides have been reported to possess multiple biological activities, including anti-osteoporotic [9], anticancer [10], anti-inflammatory [11], and antioxidant effects [12], and are considered key active constituents responsible for the pharmacological effects of velvet antler. Accordingly, antioxidant peptides derived from velvet antler hold considerable potential for further research and development due to their promising antioxidative properties.

Currently, the screening and identification of antioxidant peptides primarily rely on conventional experimental approaches, including enzymatic hydrolysis, separation and purification, in vitro antioxidant assays, and structural characterization [13]. Although several methods have been developed to identify peptides without the need for purification, these techniques are limited in their ability to provide comprehensive compositional information, particularly regarding short peptides [14]. Therefore, the cumbersome processes of separation, purification, and both in vitro and in vivo functional validation remain indispensable. These methods are often labor-intensive, time-consuming, and costly, hindering the efficient discovery and development of antioxidant peptides.

Keap1, a pivotal regulator of the oxidative stress response, serves as a critical bridge between conventional approaches and novel antioxidant peptide research. By modulating the Nrf2 signaling pathway, Keap1 senses oxidative stress and regulates antioxidant gene expression [15]. Its unique structure, comprising the N-terminal domain, Broad-complex, Tramtrack, and Bric-à-brac domain domain, Intervening Region domain, Kelch domain, and C-terminal domain, establishes it as a classic target for antioxidant peptide design [16]. The N-terminal domain, located at the protein’s N-terminus, primarily maintains Keap1’s overall structural stability, supporting other functional domains. The BTB domain mediates Keap1 dimerization and interaction with Cullin-3, forming an E3 ubiquitin ligase complex crucial for Nrf2 degradation. The IVR domain, rich in cysteine residues such as Cys151, Cys273, and Cys288, is highly sensitive to oxidative stress, acting as a sensor for redox status. The Kelch domain, composed of six Kelch repeat sequences, forms a β-propeller structure that directly binds the Neh2 domain of Nrf2, anchoring it in the hinge-and-latch model [17]. The C-terminal domain facilitates proper protein folding and stability, enhancing Keap1’s functional integration. Antioxidant peptides developed to target the Keap1-Nrf2 interaction can disrupt the hinge-and-latch mechanism, promoting Nrf2 activation and enhancing cellular antioxidant capacity, thus providing a vital direction for the efficient screening and development of novel antioxidant peptides.

In recent years, with the advancement of proteomics and artificial intelligence (AI) technologies, in silico hydrolysis combined with machine learning-based prediction models has emerged as a promising strategy for antioxidant peptide discovery, providing new technical means for the rapid identification of bioactive peptides [18,19,20]. These approaches simulate proteolytic digestion of protein sequences and use AI models to predict and screen potential antioxidant peptides, thereby reducing experimental workload and improving screening efficiency to a certain extent. Based on this strategy, the present study employed available in silico hydrolysis tools and AI-based predictive platforms to identify potential antioxidant peptides from velvet antler protein sequences, followed by experimental validation. This work seeks to establish a framework for the discovery and development of velvet antler-derived antioxidant peptides.

## 2. Materials and Methods

### 2.1. Materials

Two antioxidant peptides with 95% purity, PHPAPTL and VPHGL, were purchased from Neogene Biochemical Technology Co., Ltd. (Shenyang, China). Cell Counting Kit-8 (CCK-8), D-Galactose, Glutathione (peptide chain: GSH), and 1,1-diphenyl-2-picrylhydrazyl (DPPH) were obtained from Solarbio Science & Technology Co., Ltd. (Beijing, China).

### 2.2. In Silico Hydrolysis and Antioxidant Peptide Screening

All protein sequence data of velvet antler were retrieved from the NCBI (https://www.ncbi.nlm.nih.gov/, accessed on 25 November 2024) database [21]. The protein hydrolysis process was simulated in silico using Python (Version 3.12.4, Python Software Foundation, Wilmington, DE, USA), with pepsin and trypsin employed as the hydrolytic enzymes. ToxinPred (https://webs.iiitd.edu.in/raghava/toxinpred/index.html, accessed on 25 November 2024) was used to predict the potential toxicity of the generated peptides [22]. Antioxidant properties of the peptides were predicted using both AnOxPP (http://www.cqudfbp.net/AI-Tools/AnOxPP/index.jsp, accessed on 25 November 2024, Chongqing University, Chongqing, China) and AnOxPePred 1.0 (https://services.healthtech.dtu.dk/services/AnOxPePred-1.0/, accessed on 25 November 2024, Technical University of Denmark, Lyngby, Denmark), and potential antioxidant peptides were screened based on the prediction results [23,24]. The peptides predicted by AnOxPP with an antioxidant probability of 1 were subjected to further evaluation using AnOxPePred, which comprehensively assessed their antioxidant potential through quantitative analysis of metal chelation capacity and free radical scavenging activity. Additionally, peptides identified as toxic by ToxinPred were systematically excluded from subsequent analyses. This multi-step screening strategy integrates predictive modeling with functional validation to ensure both efficacy and biosafety of candidate antioxidant peptides.

### 2.3. DPPH Radical Scavenging Assay

The DPPH free radical scavenging assay was performed according to the method described by Yang et al. [25]. Velvet antler antioxidant peptides were prepared at concentrations of 0.1 mg/mL, 0.2 mg/mL, 0.3 mg/mL, and 0.5 mg/mL. Each sample group received 100 μL of peptide solution and 100 μL of DPPH working solution, while the blank control group received 100 μL of anhydrous ethanol and 100 μL of DPPH working solution. The mixed solutions were incubated at room temperature (25 °C) for 30 min in the dark to prevent light-induced degradation. The absorbance was measured at 517 nm using a microplate reader. The DPPH radical scavenging rate was calculated as follows:

(1) DPPH scavenging rate (%) = [1 − (As − Ac)/A] × 100

where As, Ac and A represent the absorbance values of the sample, control, and blank groups, respectively.

### 2.4. Cell Culture and Subculture

Cells were cultured in high-glucose DMEM supplemented with 10% fetal bovine serum (FBS) and 1% penicillin–streptomycin in a humidified incubator at 37 °C with 5% CO_2_. Subculture was performed every 2–3 days when cell confluence reached 80–90%. After discarding the culture medium and rinsing with PBS, cells were detached using 0.25% trypsin–EDTA. Once cells were detached, the digestion was terminated, and cells were subcultured at a ratio of 1:3. All cells used in the experiments were in the logarithmic growth phase. HepG2 cells were maintained under the same conditions.

### 2.5. CCK-8 Cell Viability Assay

Cells were seeded in 96-well plates at a density of 6 × 10^3^ cells per well and incubated in serum-free medium for 24 h. Cells were then treated with different concentrations of peptides or drugs. Blank, control, and experimental groups were set up accordingly. After the treatment, 10 μL of CCK-8 solution was added to each well and incubated at 37 °C for 1 h. Absorbance was measured at 450 nm using a microplate reader. Cell viability was calculated using the following formula:

(2) Cell viability (%) = [(As − A)/(Ac − A)] × 100

where As, Ac and A represent the absorbance values of the sample, control, and blank groups, respectively.

### 2.6. Oxidative Damage Model Construction

To establish the oxidative damage model, blank, control, and experimental groups were set up. Cells were treated with D-Galactose at final concentrations of 50, 150, 250, 350, and 450 mM. Cell viability under oxidative stress was assessed at 12 h and 24 h using the CCK-8 assay. Results indicated that at a concentration of 350 mM D-Galactose for 24 h, cell viability decreased to approximately 50%, confirming successful model establishment for subsequent experiments.

### 2.7. Protective Effects of Velvet Antler Antioxidant Peptides on D-Galactose-Induced Cytotoxicity

Peptides were prepared at concentrations of 0.1, 0.2, 0.3, and 0.5 mg/mL. Blank, control, model, and experimental groups were set up accordingly. Both the model and experimental groups were treated with D-Galactose at a final concentration of 350 mM. After 24 h of incubation, cell viability was assessed using the CCK-8 assay.

### 2.8. Molecular System Preparation

In this study, the crystal structure of Keap1 protein (PDB ID: 2FLU) was obtained from the Protein Data Bank (https://www.rcsb.org/, accessed on 23 January 2025, Research Collaboratory for Structural Bioinformatics, Rutgers University, Piscataway, NJ, USA) [26]. The three peptide structures were predicted using Avogadro (Version1.2.0n, Open Chemistry, Pittsburgh, PA, USA) [27]. Prior to molecular docking, the Keap1 structure was preprocessed in Discovery Studio 2021 by removing water molecules and ligands around the active site.

### 2.9. Molecular Docking

Molecular docking was performed using the Hpepdock (http://huanglab.phys.hust.edu.cn/hpepdock/, accessed on 24 January 2025, Hu’s Lab, Huazhong University of Science and Technology, Wuhan, China) server [28]. The docking results were visualized and analyzed using PyMOL (Version 3.0.3, Schrödinger, LLC, New York, NY, USA) and LigPlot+ (version v.2.3, European Bioinformatics Institute, Cambridge, UK) to identify the optimal binding conformations of the peptides with the protein [29,30]. The best binding pose was selected as the initial structure for subsequent molecular dynamics (MD) simulations.

### 2.10. Molecular Dynamics Simulations

MD simulations were carried out using AMBER 22 software (University of California, San Francisco, CA, USA) [31]. Three simulation systems were constructed: the apo Keap1 protein (Apo group), Keap1 bound to antioxidant peptide PHPAPTL (PHPAPTL group), and Keap1 bound to antioxidant peptide VPHGL (VPHGL group). Simulations aimed to investigate the interactions and dynamic structural changes between the antioxidant peptides and Keap1. All simulations used the AMBER ff19SB force field [32]. The systems were solvated in an OPC water model and placed in an octahedral periodic box with a 15 Å buffer [33]. Appropriate amounts of Na^+^ ions were added for charge neutralization. Long-range electrostatics were treated using the Particle Mesh Ewald (PME) method with a cutoff distance of 10 Å [34].

After system construction, initial energy minimization was performed in two stages: 6000 steps using the steepest descent method, followed by 6000 steps of conjugate gradient minimization. Systems were then gradually heated to 310 K under Number of particles, Volume, and Temperature (NVT) ensemble using a Nose–Hoover thermostat. After heating, a 200 ns equilibrium simulation was conducted for each system under NVT conditions with a 2 fs timestep. System stability was evaluated by monitoring Root Mean Square Deviation (RMSD) fluctuations of kinetic and potential energies, temperature, and other parameters. Trajectory snapshots were recorded every 0.1 ns, generating 2000 frames per simulation. AmberTools23 was used for trajectory analysis, calculating parameters such as RMSD, radius of gyration (R_g_), solvent-accessible surface area (SASA), root-mean-square fluctuation (RMSF), and secondary structure distribution (DSSP).

### 2.11. Method of Dynamic Cross-Correlation Matrix (DCCM) Analysis

DCCM analysis was performed to quantify the correlated motions between protein residues and reveal functionally related dynamic coupling networks [35]. Position time series data of all residues were extracted from the MD trajectories after removing translational and rotational motions. Normalized cross-correlation coefficients were calculated for each pair of residues i and j. Positive values indicated correlated motion, while negative values indicated anti-correlated motion. Results were visualized as heatmaps using R Studio (version 2024.12.1+563, Posit Software, PBC, Boston, MA, USA), with color intensity representing the correlation strength.

### 2.12. Molecular Mechanics Poisson–Boltzmann Surface Area (MMPBSA) Analysis

The MMPBSA method was used to calculate the binding free energy between protein and peptide from MD trajectories. The binding affinity was evaluated by decomposing the van der Waals, electrostatic, and solvation energy contributions, and key residues contributing to binding energy were identified.

### 2.13. Statistical Analysis

Statistical analysis was performed using GraphPad Prism software (version 8.0). All data represent the mean ± standard deviation of three independent experiments, with *p*-values < 0.05 considered statistically significant.

## 3. Results and Discussion

### 3.1. Screening of Deer Antler-Derived Peptides

Initially, the deer antler protein obtained from NCBI was hydrolyzed using pepsin and trypsin. The resulting peptides were subjected to antioxidant activity prediction via AnOxPP for preliminary screening. Subsequently, the antioxidant potential of the peptides was re-evaluated using AnOxPePred, combined with toxicity assessment through ToxinPred for secondary screening. As shown in Table 1, the top 10 candidates based on comprehensive antioxidant activity rankings were identified, from which the antioxidant peptides VPHGL and PHPAPTL were ultimately selected for further analysis. The selected peptides, VPHGL and PHPAPTL, were chemically synthesized in vitro. The machine learning screening results of deer antler antioxidant peptides are present in the Supplementary Materials.

### 3.2. DPPH Radical Scavenging Activity of Velvet Antler Antioxidant Peptides

**Figure 1:**
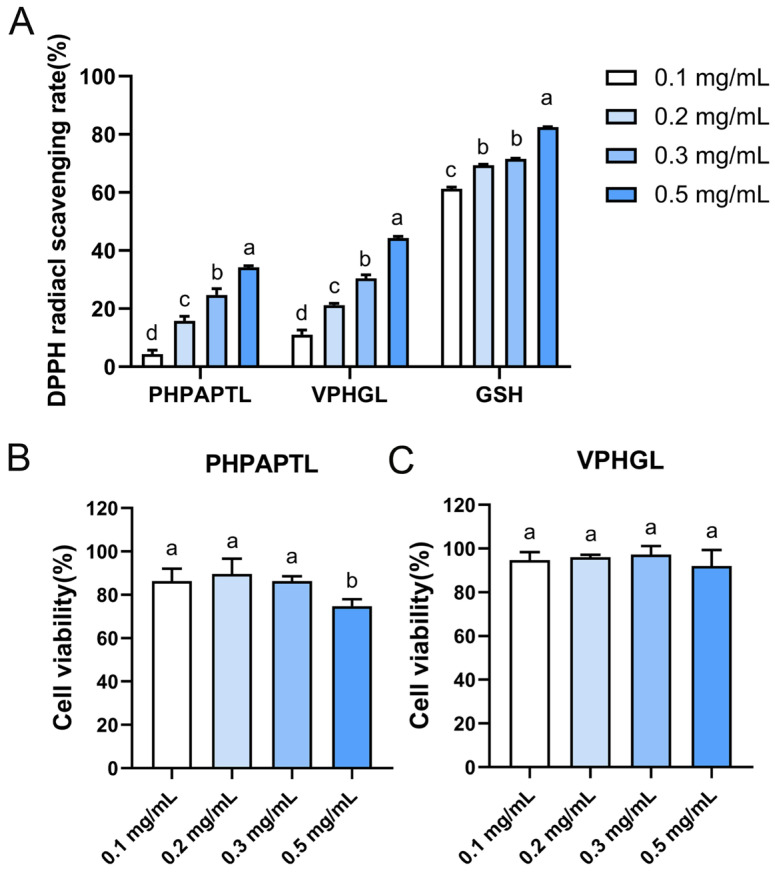
In vitro experiments on radical scavenging and cytotoxicity of the two peptides. (**A**) Radical scavenging rates of the two antioxidant peptides. (**B**) Cytotoxicity of peptide PHPAPTL at different concentrations. (**C**) Cytotoxicity of peptide VPHGL at different concentrations. Different letters (a–d) in the figure indicate significant differences (*p* < 0.05).

When DPPH radicals react with antioxidants, the radicals are scavenged, resulting in a reduction in absorbance [36]. To evaluate the antioxidant activity of velvet antler-derived peptides, the DPPH radical scavenging activity of PHPAPTL and VPHGL was determined at concentrations of 0.1, 0.2, 0.3, and 0.5 mg/mL (Figure 1). The results showed that the DPPH scavenging rates of both peptides increased with concentration. At 0.5 mg/mL, the scavenging rates of VPHGL and PHPAPTL were 44.28% and 34.17%, respectively, indicating their radical-scavenging capacity under in vitro conditions. Moreover, VPHGL exhibited significantly stronger DPPH radical scavenging activity than PHPAPTL (*p* < 0.05). In comparison, GSH (0.5 mg/mL) showed a much higher scavenging rate of 82.45%. Thus, the DPPH scavenging activities of PHPAPTL and VPHGL were relatively weaker than that of the positive control. Since the DPPH assay represents an idealized model system that differs substantially from the complex in vivo environment, the observed in vitro scavenging activities of PHPAPTL and VPHGL suggest their potential antioxidant properties, which warrant further investigation.

### 3.3. Cytotoxicity of Velvet Antler Antioxidant Peptides in HepG2 Cells

The cytotoxicity of antioxidant peptides is typically assessed by measuring cell viability [37]. In this study, the effects of four concentrations (0.1, 0.2, 0.3, and 0.5 mg/mL) of the peptides on the viability of HepG2 cells were evaluated after 24 h of incubation (Figure 1B,C). The results showed that PHPAPTL exhibited no apparent cytotoxicity at concentrations of 0.1, 0.2, and 0.3 mg/mL. However, at 0.5 mg/mL, cell viability decreased to 74.73% (*p* < 0.05), indicating cytotoxic effects at higher concentrations. This finding is inconsistent with the previous in silico toxicity prediction, which may be attributed to the greater complexity of the intracellular environment. Once inside the cell, the peptide may undergo unexpected interactions or reactions that contribute to cytotoxicity, thereby exceeding the predictive capacity of the computational algorithm. In contrast, VPHGL showed no significant effect on HepG2 cell viability at any tested concentration.

### 3.4. Protective Effects of Antioxidant Peptides Against D-Galactose-Induced Oxidative Stress

**Figure 2:**
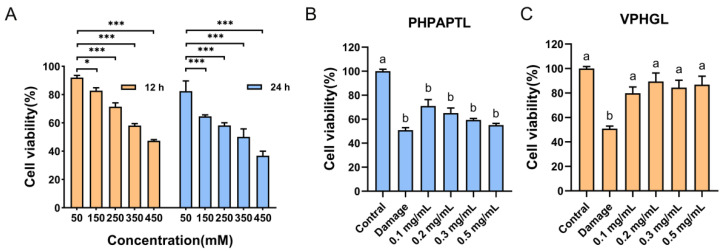
Impact of D-galactose and peptides (PHPAPTL, VPHGL) on HepG2 cell viability. (**A**) Effect of D-galactose on the viability of HepG2 cells. “*” indicates *p* < 0.05, “***” indicates *p* < 0.001. Effects of PHPAPTL (**B**) and VPHGL (**C**) on cell viability under D-galactose-induced oxidative stress. Different letters (a–b) in the figure indicate significant differences (*p* < 0.05).

D-galactose (D-Gal) is metabolized primarily by galactose oxidase in vivo, and abnormal increases in intracellular D-Gal levels can significantly inhibit antioxidant enzyme activity, leading to oxidative stress [38]. As the concentration and exposure time of D-Gal increased, HepG2 cell viability progressively declined. To establish a suitable oxidative damage model, cell viability was maintained at approximately 50%, which balances injury severity with cell survival [39]. The results revealed that treatment with 350 mM D-Gal for 24 h reduced HepG2 cell viability to 50.12% (Figure 2), confirming successful model establishment.

The protective effects of the two antioxidant peptides against D-Gal-induced oxidative damage were subsequently evaluated (Figure 2). PHPAPTL at 0.1 mg/mL significantly increased cell viability compared with the model group (*p* < 0.05). However, the protective effect diminished with increasing concentrations, and at 0.5 mg/mL, no significant difference was observed relative to the model group, which might be attributed to its cytotoxicity at high concentrations. In contrast, VPHGL at all tested concentrations significantly enhanced cell viability (*p* < 0.05) compared with the model group, indicating a good protective effect against oxidative stress-induced cell injury.

### 3.5. Molecular Docking Analysis

**Figure 3:**
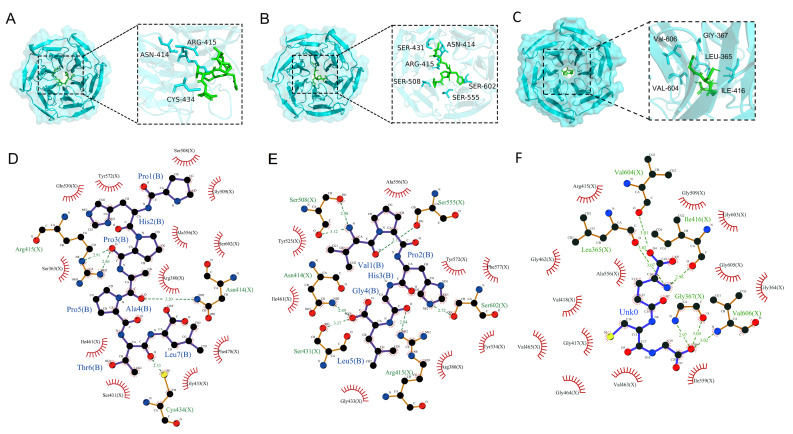
Molecular docking results of the two peptides with KEAP1. (**A**) Docking results of PHPAPTL with KEAP1. (**B**) Docking results of VPHGL with KEAP1. (**C**) Docking results of GSH with KEAP1. (**D**) Interaction analysis between PHPAPTL and KEAP1. (**E**) Interaction analysis between VPHGL and KEAP1. (**F**) Interaction analysis between GSH and KEAP1. In D–F, green dashed lines represent hydrogen bonds.

Molecular docking was performed to evaluate the interactions between the antioxidant peptides and the Keap1 protein, with results presented in (Figure 3). The peptides were highlighted in green, and interacting amino acid residues were labeled in blue to distinguish key interaction sites. Hydrogen bonding was identified as a major interaction type. PHPAPTL formed hydrogen bonds with residues ARG-415, ASN-414, and CYS-434, while VPHGL interacted via hydrogen bonds with ASN-414, SER-431, ARG-415, SER-508, SER-602, and SER-555. These residues were considered important contributors to the peptide-Keap1 binding process (Figure 3C,D). Hydrophobic interactions between the peptides and residues Gly443, Arg380, Ile461, Ala556, and Tyr572, along with recurring hydrogen bonds involving Arg415 and Asn414, suggest these residues are critical for peptide-Keap1 binding.

### 3.6. Structural Stability Analysis

**Figure 4:**
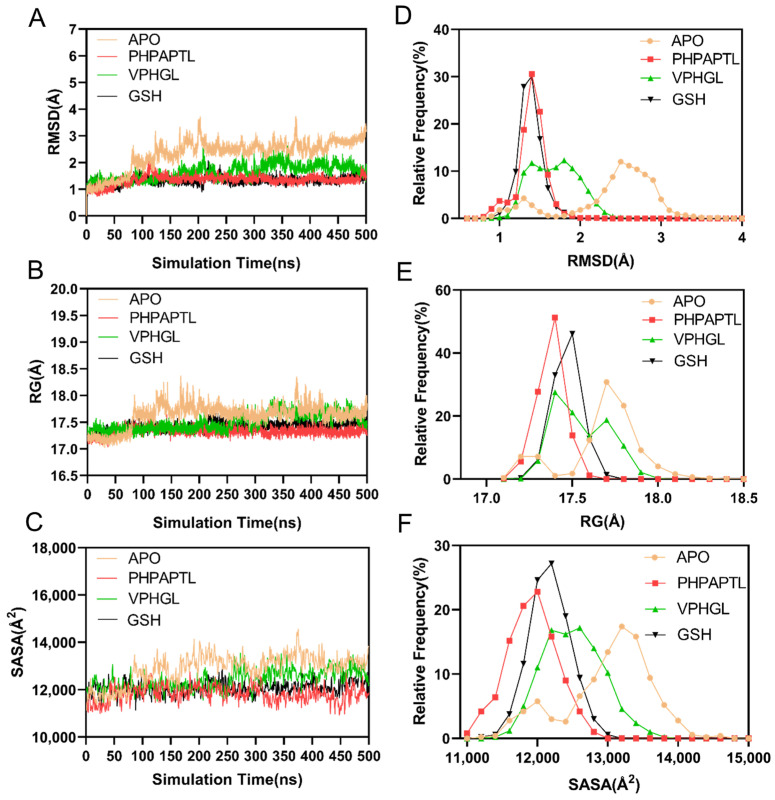
Analysis of structural stability. (**A**) The temporal evolution of the RMSD from their initial structure of the Apo, PHPAPTL, VPHGL and GSH systems. (**B**) The temporal evolution of the R_g_ from their initial structure of the Apo, PHPAPTL, VPHGL and GSH systems (**C**) The temporal evolution of the SASA from their initial structure of the Apo, PHPAPTL, VPHGL and GSH systems (**D**) Distribution of RMSD values in the four systems. (**E**) Distribution of R_g_ values in the four systems. (**F**) Distribution of SASA values in the four systems.

The root-mean-square deviation (RMSD) of C_α_ atoms was calculated to assess system stability. As shown in Figure 4A,D, all systems stabilized after 200 ns. The average RMSD values for the Apo, VPHGL, GSH, and PHPAPTL systems were 2.34 Å, 1.63 Å, 1.33 Å, and 1.34 Å, respectively, indicating enhanced stability upon peptide binding.

The radius of gyration (R_g_) analysis (Figure 4B,E) revealed structural compactness. The mean R_g_ values for Apo, VPHGL, GSH, and PHPAPTL were 17.63 Å, 17.41 Å, 17.42 Å, and 17.33 Å, respectively, consistent with the RMSD results. The Solvent Accessible Surface Area (SASA) is the surface area of a biomolecule (such as a protein) that is accessible to solvent molecules (like water), typically measured in square angstroms, used to assess protein folding, interactions, and function. Similarly, solvent-accessible surface area (SASA) trends (Figure 4C,F) showed average values of 12,893.59 Å^2^, 12,406.21 Å^2^, 12,077.09 Å^2^, and 11,821.19 Å^2^ for the three systems, with the Apo system exhibiting the largest fluctuations and GSH the smallest.

**Figure 5:**
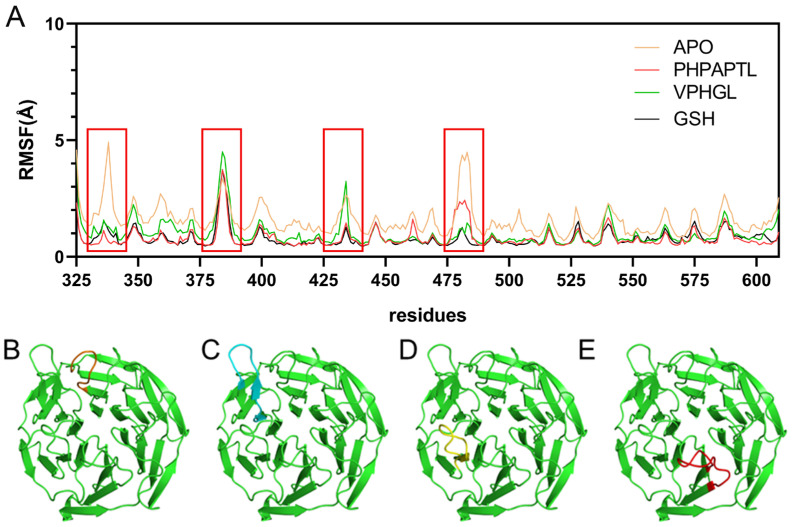
RMSF Results from Molecular Dynamics Simulations. (**A**) RMSF diagrams for the four systems. The red box highlights residues with higher fluctuations, corresponding to the regions shown in Figures B–E. (**B**) Structure of residues 330-345 in orange. (**C**) Structure of residues 376–390 in cyan. (**D**) Structure of residues 425-438 in yellow. (**E**) Structure of residues 474-488 in red.

The root mean square fluctuation (RMSF) of C_α_ atoms was calculated to evaluate residue-specific flexibility across the simulated systems, as depicted in Figure 5. In the Apo system, elevated RMSF values were observed at residues 330–340 and 475–485, indicating pronounced conformational flexibility in these regions. Notably, these fluctuations were significantly attenuated upon binding of VPHGL, GSH or PHPAPTL, suggesting that peptide interaction restricts dynamic motions in these segments. Conversely, residues 380–390 and 430–440 exhibited increased RMSF values in the peptide-bound systems compared to the Apo state. This enhanced flexibility in peptide-bound systems implies that these regions may undergo structural rearrangements critical for mediating the inhibitory effects of the peptides on Keap1. The contrasting RMSF profiles between bound and unbound states highlight localized changes in protein dynamics induced by peptide binding, potentially linking these residue-specific motions to functional modulation of Keap1 activity.

### 3.7. MM/PBSA Analysis

**Figure 6:**
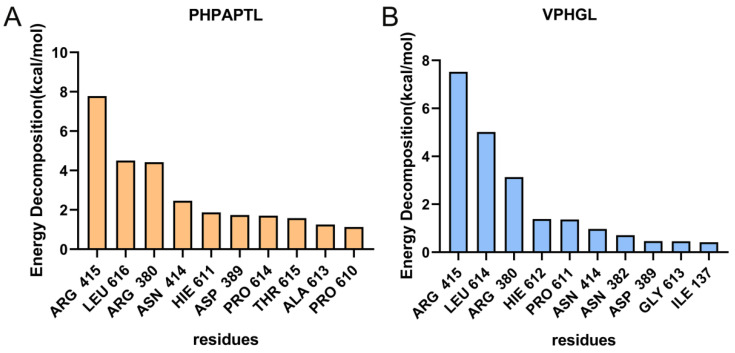
Contributions of amino acid residues to binding free energy in the (**A**) PHPAPTL and (**B**) VPHGL systems.

MM/PBSA (Molecular Mechanics Poisson-Boltzmann Surface Area) analysis was conducted to identify key residues involved in the binding of the antioxidant peptides to Keap1. As shown in Figure 6, residues Arg415, Arg380, Asn414, and Asp389 were identified as critical contributors to the binding energy in both peptide-Keap1 complexes. In addition, the leucine residues at positions Leu616 and Leu614 may play a functional role. The binding free energy values summarized in Table 2 revealed that PHPAPTL exhibited a stronger binding affinity compared to VPHGL, suggesting its superior stability within the Keap1 binding pocket.

### 3.8. Dynamic Cross-Correlation Matrix (DCCM) Analysis

**Figure 7:**
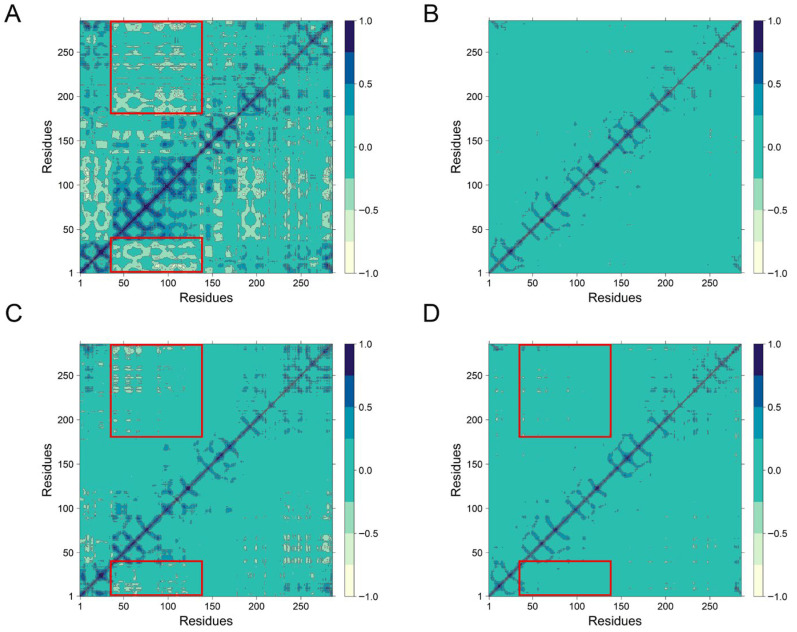
The dynamical cross-correlation matrix diagrams of (**A**) Apo, (**B**) PHPAPTL, (**C**) VPHGL and (**D**) GSH systems. (The large red box contains residues 363–366, and the small red box contains 452–609).

To further investigate the impact of peptide binding on residue motion dynamics, dynamic cross-correlation matrices (DCCM) were generated for all systems (Figure 7). In the Apo system (Figure 7A), residues 40–140 displayed pronounced long-range anti-correlated motions (indicated by blue regions), reflecting dynamic flexibility in the unbound state. These anti-correlations were partially attenuated in the VPHGL-bound system. In contrast, the PHPAPTL and GSH bound system exhibited minor displacement of residues, as evidenced by reduced anti-correlated motions, likely due to the tight and stable binding of the peptide to Keap1, which restricts residue mobility.

### 3.9. Secondary Structure Analysis

**Figure 8:**
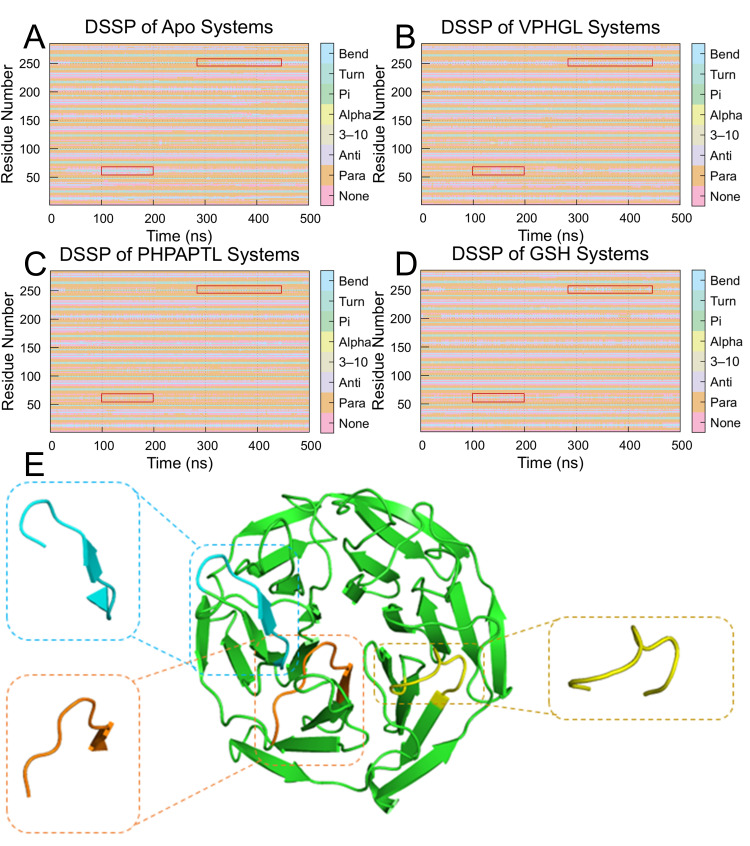
Secondary structure transition probabilities for residues 325–609 in the (**A**) Apo, (**B**) VPHGL, (**C**) PHPAPTL and (**D**) GSH systems. The red box highlights regions with secondary structure changes distinct from the apo protein. (**E**) Structural conformations of residues 377–385, 411–419, and 479–487 upon binding to Keap1.

Changes in the secondary structure of Keap1 during the simulations were analyzed using DSSP (Dictionary of Protein Secondary Structure). As illustrated in Figure 8, peptide binding significantly altered the structural dynamics of Keap1. Specifically, the frequency of transitions between Bend and Turn conformations decreased at residues 380–390 and 550–560 in the peptide-bound systems compared to the Apo system. In the Apo system, frequent structural shifts between Bend and Turn were observed near residues Arg483, Arg380, and Ser555, with occasional coexistence of two Bend or Turn motifs. However, PHPAPTL stabilized these regions, maintaining a single Bend or Turn conformation throughout the simulation, while VPHGL and GSH retained dual conformations. Furthermore, an increase in parallel β-sheet content was detected in the peptide-bound systems, suggesting that β-sheet formation may play a role in stabilizing the peptide-Keap1 interaction.

### 3.10. Prediction of Peptide Properties

To further assess the drug-likeness potential of peptides, we utilized the ADMET-AI (https://admet.ai.greenstonebio.com/, accessed on 18 September 2025, Greenstone Biosciences, Stanford University, Stanford, CA, USA) tool to systematically predict their properties, including drug-likeness (QED), oral bioavailability, and human intestinal absorption (HIA) (results shown in Table 3) [40]. The predictions reveal that the pentapeptide VPHGL outperforms the tripeptide glutathione (GSH) in two critical metrics—drug-likeness (QED) and human intestinal absorption (HIA)—demonstrating superior developmental potential and oral absorption characteristics.

Although the model predicts a higher oral bioavailability for GSH compared to VPHGL, its practical applicability may be limited. This is primarily due to GSH’s smaller, more flexible tripeptide structure, which is less likely to form stable secondary structures. Consequently, GSH is more susceptible to hydrolysis by proteases in the gastrointestinal tract, leading to rapid degradation. Such complex in vivo biological factors are often overlooked in current computational prediction models. Therefore, the high oral bioavailability predicted for GSH should be interpreted cautiously in light of its chemical properties.

## 4. Conclusions

This study successfully identified and characterized two antioxidant peptides, PHPAPTL and VPHGL, from deer antler hydrolysates. Free radical scavenging assays demonstrated scavenging rates of 34.17% for PHPAPTL and 44.28% for VPHGL. In vitro experiments on HepG2 cells revealed that PHPAPTL significantly enhanced cell viability at 0.1 mg/mL compared to the injury model group, though viability declined at higher concentrations. In contrast, VPHGL maintained elevated cell viability across all tested concentrations. Molecular docking results highlighted robust interactions between both peptides and Keap1, with recurring hydrogen bonds involving Arg415 and Asn414. Molecular dynamics simulations further confirmed the stability of these complexes, showing reduced RMSD and R_g_ values in peptide-bound systems. Notably, peptide binding induced dynamic structural rearrangements near the active site, including suppressed Bend/Turn transitions and increased β-sheet content. These findings not only validate the strong binding of PHPAPTL and VPHGL to Keap1 but also underscore their potential as potent antioxidants, providing a foundation for future development of peptide-based antioxidant therapeutics. In comparison, although PHPAPTL demonstrated favorable results in molecular dynamics simulations, its mild toxicity limits its antioxidant performance, highlighting the need for more stringent screening criteria. In contrast, VPHGL exhibited promising results in both molecular dynamics simulations and property predictions, indicating significant potential and warranting further analysis. Future efforts may focus on optimizing peptide sequences to enhance stability, applying chemical modifications to extend half-life, improving bioavailability through delivery systems, and validating efficacy in more physiologically relevant models.

## Figures and Tables

**Table 1:** The top 10 candidate peptides identified by AnOxPePred and ToxinPred3.0.

NO.	Sequence	Scavenger	Chelator	Score	Toxicity Prediction	Toxicity Likelihood
1	PPPPL	0.53	0.34	0.47	Toxic	0.69
2	VPHGL	0.54	0.29	0.46	Non-Toxic	0.25
3	CAPHPL	0.53	0.30	0.46	Toxic	0.74
4	QQPPPAPL	0.50	0.31	0.44	Toxic	0.43
5	EPAHL	0.50	0.30	0.44	Non-Toxic	0.16
6	PHPAPTL	0.49	0.30	0.44	Non-Toxic	0.30
7	ANTPHL	0.50	0.28	0.43	Non-Toxic	0.17
8	PGEPGL	0.51	0.26	0.43	Non-Toxic	0.25
9	PPTGIHPL	0.51	0.25	0.43	Toxic	0.48
10	GGDGNHVL	0.51	0.24	0.43	Non-Toxic	0.16

**Table 2:** Comparative MMPBSA Analysis of Ligand-Protein Binding Energetics and Stability.

System	PHPAPTL	VPHGL	GSH
ΔE_vdw_	−29.03 ± 0.73	−18.60 ± 0.48	−30.90 ± 0.23
ΔE_ele_	−183.62 ± 3.56	−161.46 ± 2.63	−53.73 ± 1.11
ΔG_solv_	182.76 ± 3.25	154.95 ± 2.54	85.95 ± 0.98
ΔG_gas_	−212.65 ± 3.84	−180.05 ± 2.75	−89.63 ± 1.16
ΔG_total_	−29.89 ± 0.80	−25.10 ± 0.48	−3.68 ± 0.64

**Table 3:** Prediction results of peptide properties.

Peptide	Quantitative Estimate of Druglikeness	Human Intestinal Absorption	Oral Bioavailability
PHPAPTL	0.09	0.09	0.23
VPHGL	0.19	0.45	0.49
GSH	0.13	0.25	0.61

## Data Availability

The original contributions presented in this study are included in the article. Further inquiries can be directed to the corresponding author(s).

## Supplementary Materials

The following supporting information can be downloaded at: https://www.mdpi.com/article/10.3390/antiox14101169/s1.

## Abbreviations

The following abbreviations are used in this manuscript:

D-Gal	D-galactose
ROS	reactive oxygen species
AI	artificial intelligence
CCK-8	Counting Kit-8
DPPH	1,1-diphenyl-2-picrylhydrazyl
FBS	fetal bovine serum
R_g_	radius of gyration
SASA	solvent-accessible surface area
RMSF	root-mean-square fluctuation
DSSP	secondary structure distribution
DCCM	dynamic cross-correlation matrices
Keap1	Kelch-like ECH-associated protein 1
